# Supplementary material for: Cardiovascular Complications of COVID-19 among Pregnant Women and Their Fetuses: A Systematic Review
Source: J Clin Med. 2022 Oct 20;11(20):6194. doi: 10.3390/jcm11206194 (PMC9604883; doi:10.3390/jcm11206194)
Supplement: Supplementary file 1 [file jcm-11-06194-s001.zip › jcm-1949850-supplementary.pdf]

Table S1. Quality assessment of the included cohort, case-control, (and cross-sectional) studies

Author (ref.)	selection				comparability	outcome			Total score	Total Quality
	1	2	3	4	1	1	2	3		
Adhikari et al. [31]	*	*	*	*	*	*	*	*	8	Good
Anuk et al. [32]	*	*	*	*	*	*	*		7	Good
Wu Y [33]	*	*	*	*		*	*	*	7	Fair
Pachtman et al. [34]	*	*	*	*		*	*	*	7	Fair
Berengueet et al. [35]	*	*	*	*		*	*	*	7	Fair
Brandt et al. [36]	*	*	*	*	**	*	*	*	9	Good
Jering et al. [37]	*	*	*	*	*	*	*	*	8	Good
Soto-Torres et al. [38]	*	*	*	*	**	*	*	*	9	Good
Sule et al. [42]	*	*	*		*	*	*	*	7	Good
Rosenbloom et al. [39]	*	*	*		**	*	*		7	Good
Mahajan et al. [15]	*	*	*		*	*	*	*	7	Good
Melguizo et al. [16]	*	*	*	*	*	*	*	*	8	Good
Mendoza et al. [17]	*	*	*	*	**	*	*	*	9	Good
Mercedes et al. [18]	*	*	*		**	*	*	*	8	Good
Molteni et al. [19]	*	*	*		*	*	*	*	7	Good
Mullins et al. [20]	*	*	*	*	*	*	*	*	8	Good
Chavan et al. [21]	*				**	**			5	Satisfactory
Osaikhuwuomwan et al. [22]	*		*	**	**	**			8	Good
Palomo et al. [23]	*	*	*		**	*	*	*	8	Good
Papageorghiou et al. [24]	*	*	*		**	*	*	*	8	Good
Pierce-Williams et al. [25]	*	*	*	*	**	*	*	*	9	Good
Pirjani et al. [26]	*	*	*		*	*	*	*	7	Good
Rajan et al. [27]	*	*	*		**	*	*		7	Good
Serrano et al. [28]	*			**	**	**	*		8	Good
Sinaci et al. **[41]**	*	*	*		*	*	*	*	7	Good
Trilla et al. [29]	*	*	*		*	*	*	*	7	Good
Abedzadeh kalahroudi[1]	*	*	*		**	*	*		7	Good
Antoun et al[2]	*	*	*	*	**	*	*		8	Good
Ahlberg et al[14]	*	*	*		**	*	*		7	Good
Arslan et al[3]	*	*	*	*	**	*	*	*	9	Good
Bachani et al[4]	*	*	*	*	*	*	*		7	Fair
Cruz et al[5]	*	*	*	*	**	*	*		8	Good

Dalla et al[6]	*	*	*	*	**	*	*		8	Good
Epelboin et al[7]	*	*	*		**	*	*	*	8	Good
Ferrera et al[8]	*	*	*	*	*	*	*		7	Good
Guida et al[9]	*	*	*		**	*	*		7	Good
Gurol et al[10]	*	*	*		**	*	*	*	8	Good
Hill et al[11]	*	*	*	*	*	*	*		7	Good
Jayaram et al[12]	*	*	*	*	**	*	*		8	Good
Knobel et al[13]	*	*	*		**	*	*		7	Good
Goncu Ayhan et al**[40]**	*	*	*		**	*	*		7	Good

Table S2. Quality assessment of the included case series, and case report studies

.study	Were patient's demographic characteristics clearly described?	Was the patient's history clearly described and presented as a timeline?	Was the current clinical condition of the patient on presentation clearly described?	Were diagnostic tests or assessment methods and the results clearly described?	Was the intervention[s] or treatment procedure[s] clearly described?	Was the post-intervention clinical condition clearly described?	Were adverse events [harms] or unanticipated events identified and described?	Does the case report provide takeaway lessons?
Agarwal et al. [65]	yes	no	yes	yes	yes	yes	yes	unclear
Bhattacharyya et al. [66]	yes	yes	yes	yes	yes	yes	yes	unclear
De Vita et al. [67]	yes	unclear	yes	yes	yes	yes	yes	yes
Juusela et al. [68]	yes	yes	yes	yes	yes	yes	yes	unclear
Mongula et al. [69]	yes	yes	yes	yes	yes	yes	yes	yes
Wang et al. [74]	yes	unclear	yes	yes	yes	yes	yes	no
Leal et al.**[56]**	yes	no	unclear	yes	unclear	no	yes	unclear
Mevada et al. **[73]**	Yes	Yes	Unclear	Yes	Yes	Yes	Yes	Unclear
Naeh et al. **[57]**	Yes	Yes	Yes	Yes	Yes	Yes	Unclear	yes
Nejadrahim et al. **[58]**	Yes	Yes	Yes	Yes	Yes	Yes	Unclear	Yes
Pelayo et al. **[59]**	Yes	Yes	Yes	Yes	Yes	Yes	Unclear	yes

Radoi et al. **[60]**	Yes	Yes	Yes	Yes	Yes	Yes	Unclear	yes
Rajan et al. [27]	Yes	No	Unclear	Yes	No	Unclear	Unclear	yes
Soofi et al. **[61]**	Yes	Yes	Yes	Yes	Yes	Yes	Yes	unclear
Stout et al. **[62]**	Yes	Yes	Yes	Yes	Yes	Yes	Yes	unclear
Vaezi et al. **[63]**	yes	No	No	Unclear	Unclear	Unclear	Unclear	yes
Yousefzade et al. [30]	Unclear	No	yes	yes	unclear	unclear	unclear	yes
Zarrintan et al. **[64]**	Yes	yes	unclear	yes	yes	yes	Unclear	yes
Ahmed et al**[43]**	yes	no	yes	yes	yes	yes	yes	unclear
Askary et al**[44]**	yes	unclear	yes	yes	yes	yes	yes	yes
Azarkish et al**[45]**	yes	yes	yes	yes	yes	yes	yes	unclear
Breslin et al**[46]**	yes	yes	yes	yes	yes	yes	yes	yes
Dehghan et al**[47]**	yes	yes	yes	yes	yes	yes	yes	yes
Cetera et al**[48]**	yes	unclear	yes	yes	yes	yes	yes	unclear
Donovan et al**[49]**	yes	unclear	yes	yes	yes	yes	yes	yes
Franca et al**[50]**	yes	yes	yes	yes	yes	yes	yes	unclear
Gara et al**[51]**	yes	unclear	yes	yes	yes	yes	yes	unclear
Goudarzi et al**[52]**	yes	no	yes	yes	yes	yes	yes	no
Gulersen et al**[53]**	yes	unclear	yes	yes	yes	yes	yes	unclear
Hansen et al**[54]**	yes	no	yes	yes	yes	yes	yes	unclear
Khodamoradi et al**[55]**	yes	no	yes	yes	yes	yes	yes	yes
Gracia perez et al**[70]**	yes	yes	yes	yes	yes	yes	yes	yes
Gubbar et al**[71]**	yes	unclear	yes	yes	yes	yes	yes	no
Kato et al**[72]**	yes	no	yes	yes	yes	yes	yes	unclear
